# The Influence of NaClO on the Biocorrosion of Carbon Steel Induced by *Chlorella vulgaris* in Artificial Seawater

**DOI:** 10.3390/molecules30173636

**Published:** 2025-09-05

**Authors:** Junnan Zhang, Qi Fu, Guang-Ling Song

**Affiliations:** 1 Department of Ocean Science and Engineering, Southern University of Science and Technology, Shenzhen 518055, China; 12332228@mail.sustech.edu.cn; 2 Division of Materials Engineering, School of Mechanical and Mining Engineering, The University of Queensland, St Lucia, QLD 4072, Australia

**Keywords:** microbiologically influenced corrosion, *Chlorella vulgaris*, carbon steel, algaecide, NaClO

## Abstract

Microbiologically influenced corrosion (MIC) poses a significant threat to carbon steel facilities in marine environments. Due to its environmental friendliness and excellent bactericidal effect, NaClO has been widely applied in the marine industry to inhibit MIC. In fact, algae can also cause severe biocorrosion to carbon steels. However, there are very few studies on the biocorrosion induced by algae, and thus the algicidal effect of bactericide NaClO is still unclear. In this study, the biocorrosion of 45# mild steel induced by *Chlorella vulgaris* (*C. vulgaris*) and the effect of NaClO on the biocorrosion were systematically investigated. The results showed that the corrosion rate of the steel in *C. vulgaris*-containing biotic artificial seawater was significantly higher than that in the abiotic solution. An increase in NaClO concentration resulted in a higher corrosion rate of the steel in general but relatively mild local corrosion penetration. The overall corrosion damage of the steel in the biofilm-covered areas was alleviated, while the corrosion penetration in the biofilm-discontinuous area became deeper after NaClO addition. The addition of 1 ppm NaClO into the biotic artificial seawater could not significantly inhibit the growth of *C. vulgaris*. When NaClO concentration increased to 10 ppm, the growth of *C. vulgaris* was markedly suppressed, resulting in a lower corrosion rate than that at 0 ppm and 1 ppm NaClO. At 100 ppm of NaClO, *C. vulgaris* cells were completely killed, and the overall corrosion rate in the biotic solution was close to that in the abiotic solution. Based on the experimental observations, algae-induced corrosion and its inhibition by NaClO were finally analyzed.

## 1. Introduction

Marine corrosion causes enormous global economic losses annually. In the marine industry, carbon steels are a critical material in many applications, such as ships, platforms, and pipelines, facing severe corrosion challenges [1,2,3,4]. Existing studies have confirmed that the corrosion processes of carbon steels in marine environments are closely related to microorganisms that can adhere to the surfaces of carbon steels to form biofilms. Such accelerated corrosion is well-known as microbiologically influenced corrosion (MIC) [5,6,7,8]. At present, there are many studies on the biocorrosion of carbon steel caused by bacteria, and several mechanisms have been proposed to explain the corrosion damage, such as the cathodic depolarization theory and biocatalytic cathodic reduction theory [9,10]. These basic understandings have formed an important foundation for current corrosion mitigation strategies in marine engineering.

In marine ecosystems, the actively growing microalgae may also affect the corrosion process [11]. However, the effect of the widely available microalgae on corrosion has never been systematically studied. Recently, with increasing human activities in coastal cities and the greenhouse effect, coastal eutrophication and seawater warming have become more and more significant, which can aggravate the occurrence of algal blooms and promote the colonization of microalgae on metal surfaces [12,13,14]. Therefore, it is important to investigate the biocorrosion induced by algae and explore its mitigation measures.

*Chlorella vulgaris* (*C. vulgaris*), one of the most common photosynthetic microalgae in marine environments, exhibits remarkable environmental adaptability and rapid proliferation, making it a model microorganism in antifouling research [15,16,17]. However, studies on *C. vulgaris*-induced biocorrosion so far have been very limited. Liu et al. found that *C. vulgaris* could accelerate the corrosion of Q235 carbon steel and 316L stainless steel due to its metabolic activity and the secretion of extracellular polymeric substances (EPS) [18]. In contrast, Chen et al. reported [19] that the abundant EPS produced by the *C. vulgaris* adhering to carbon steel surfaces might act as a protective barrier to inhibit corrosion propagation. These conflicting findings suggest that the corrosion mechanism of *C. vulgaris* on carbon steel remains controversial and needs further investigation.

Sodium hypochlorite (NaClO) is an oxidizing biocide widely used in swimming pool sanitation, industrial cooling systems, medical wastewater treatment, and marine engineering due to its excellent antimicrobial properties [20,21,22,23]. However, its strong oxidizing capability simultaneously accelerates material degradation. Liu et al. [24] investigated the effect of active chlorine on the corrosion behavior of low-alloy marine steel and found that when active chlorine concentration increased from 1 ppm to 100 ppm, the corrosion rate of mild steel significantly increased, while its tensile and yield strengths remained unaffected. Su et al. [25] discovered that in sterile cooling water, 8 mg/L NaClO accelerated the corrosion of carbon steel, although its bactericidal effect indirectly mitigated MIC. Oliveira et al. [26] used 1.0 ppm NaClO + 1.0 ppm xanthan gum to control MIC in turbulent seawater, which reduced the corrosion rate of carbon steel by 45% to 62%. However, the corrosion inhibition effect was confused by the use of xanthan gum, and the concentration effect of NaClO on the microalgae-induced biocorrosion of carbon steels remains unclear. It is a straightforward speculation that an insufficient concentration of NaClO may fail to effectively inhibit algal growth, thereby reducing its efficacy in controlling the biocorrosion of carbon steel. Conversely, an excessively high concentration of NaClO may accelerate the corrosion of carbon steel, incur additional cost, and certainly cause an adverse environmental impact. It is also possible that NaClO may change the physiological characteristics of microalgae or alter the interaction mechanism of NaClO with the steel surface as the concentration of NaClO varies. More specifically, NaClO may even influence the adhesion capacity of microalgae and the composition and quantity of their metabolic byproducts, thereby indirectly altering the corrosion process of carbon steels. These unknowns must be dealt with before NaClO can find more effective and wider applications in marine engineering.

So far, the mechanism of *C. vulgaris*-induced corrosion and the NaClO inhibition effect on the growth and metabolism of *C. vulgaris* have not been clearly revealed, let alone the understanding of NaClO influence on the biocorrosion induced by *C. vulgaris*. Therefore, this study systematically investigated the effect of NaClO concentration on *C. vulgaris*-mediated biocorrosion of carbon steel, aiming to clarify the biocorrosion mechanism of mild steel induced by *C. vulgaris* and the algacidal effect of NaClO on *C. vulgaris*. The research will provide a scientific basis and technical support for MIC prevention of carbon steel structures in marine applications.

## 2. Results and Discussion

### 2.1. Growth Curve of C. vulgaris and Physicochemical Properties of Test Solutions

**Figure 1:**
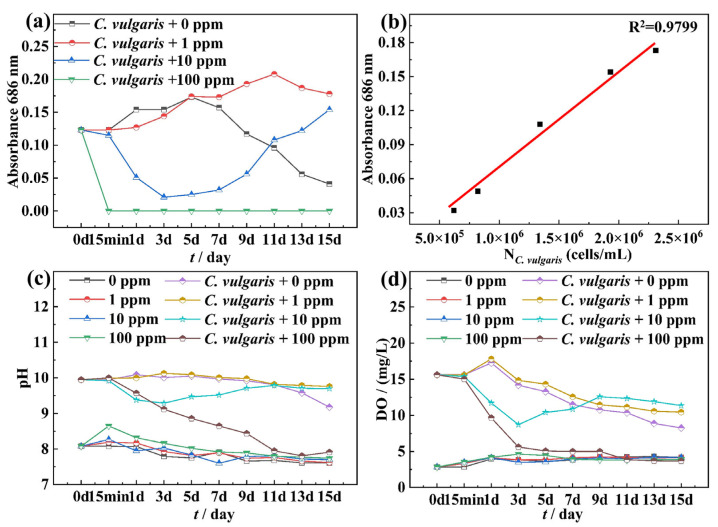
The (**a**) OD value of the biotic F/2 solution containing *C. vulgaris*, (**b**) the corresponding relation between the absorbance and the concentration of *C. vulgaris* cells in the biotic F/2 solution, (**c**) the change in pH value in the abiotic seawater and biotic F/2 solution, and (**d**) DO concentration with time in the abiotic seawater and biotic F/2 solution.

Figure 1a shows the time-dependent optical density (OD) curves of biotic F/2 solution containing *C. vulgaris* under different NaClO concentrations, which partially reflects the variation trend of *C. vulgaris* cell density. This correlation is confirmed by Figure 1b, which demonstrates a strong linear relationship (R^2^ = 0.9799) between the absorbance of the test solution and the concentration of *C. vulgaris* cells (N*_C. vulgaris_*). From Figure 1a, it can be observed that without NaClO, the concentration of *C. vulgaris* cells reached a peak on the 5th day and then gradually decreased. The 1 ppm NaClO inhibited the growth of *C. vulgaris* in the first three days and delayed the peak of *C. vulgaris* concentration to the 11th day. The 10 ppm NaClO caused a rapid decrease in the number of *C. vulgaris* cells within the first three days, followed by recovery to normal levels. When exposed to 100 ppm NaClO, *C. vulgaris* cells were completely eradicated with no viable cells detected. Figure 1c,d reveals that under 0 ppm and 1 ppm NaClO conditions, the pH and DO values of the biotic solutions showed a downward trend over time. For the 10 ppm NaClO, the time distribution characteristics of pH and DO in the biotic solution were similar to the cell concentration curve (Figure 1a), initially decreasing and then recovering to normal levels. At 100 ppm NaClO, *C. vulgaris* died completely, and pH and DO values presented a continuous downward trend over time. Comparing the 0 ppm NaClO in the abiotic and biotic solutions, it can be found that *C. vulgaris* gave rise to a significant increase in the pH of the F/2 solution, while 100 ppm NaClO completely killed *C. vulgaris*, leading to a rapid decrease in pH. Notably, in the absence of *C. vulgaris*, the addition of NaClO had no obvious effect on pH values and DO concentrations, which remained stable throughout the experimental period. 

### 2.2. Electrochemical Measurements

**Figure 2:**
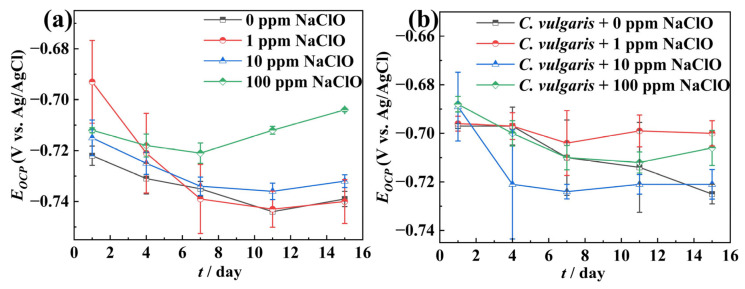
The change in the OCP over time in the abiotic solution (**a**) and the biotic solution (**b**) during the 15-day experimental period.

**Figure 3:**
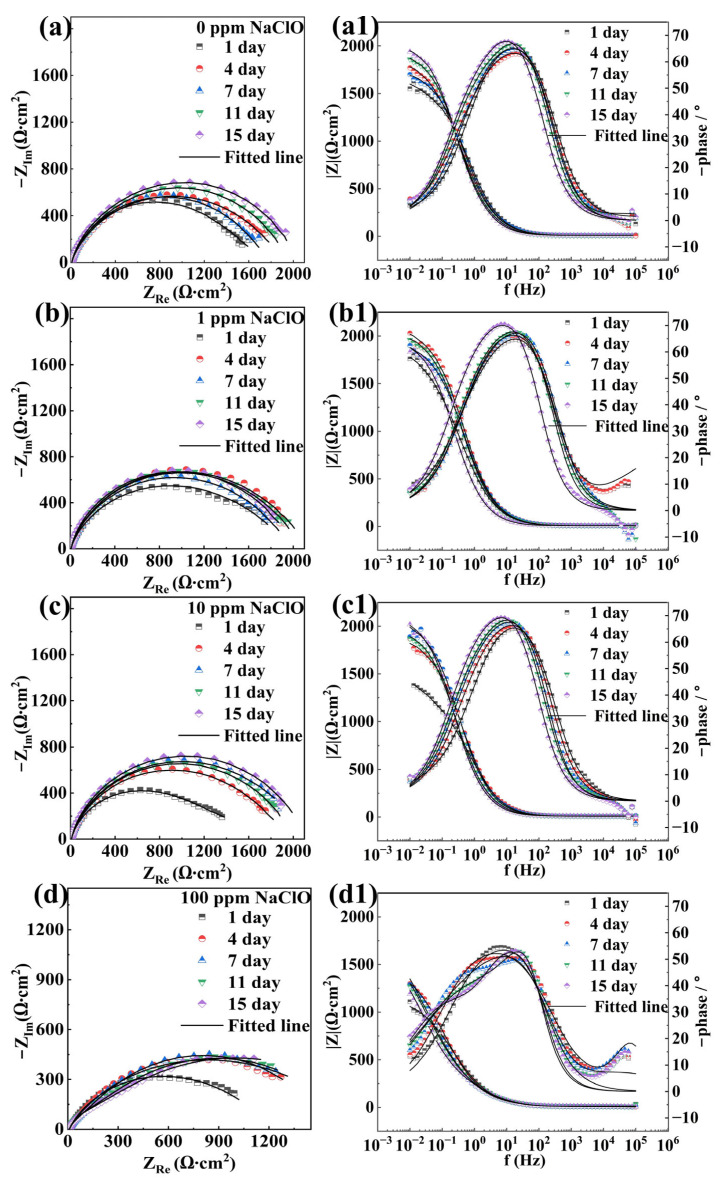
Nyquist and Bode diagrams of the specimens in the abiotic solution under the condition of (**a**,**a1**) 0 ppm, (**b**,**b1**) 1 ppm, (**c**,**c1**) 10 ppm, and (**d**,**d1**) 100 ppm NaClO.

**Figure 4:**
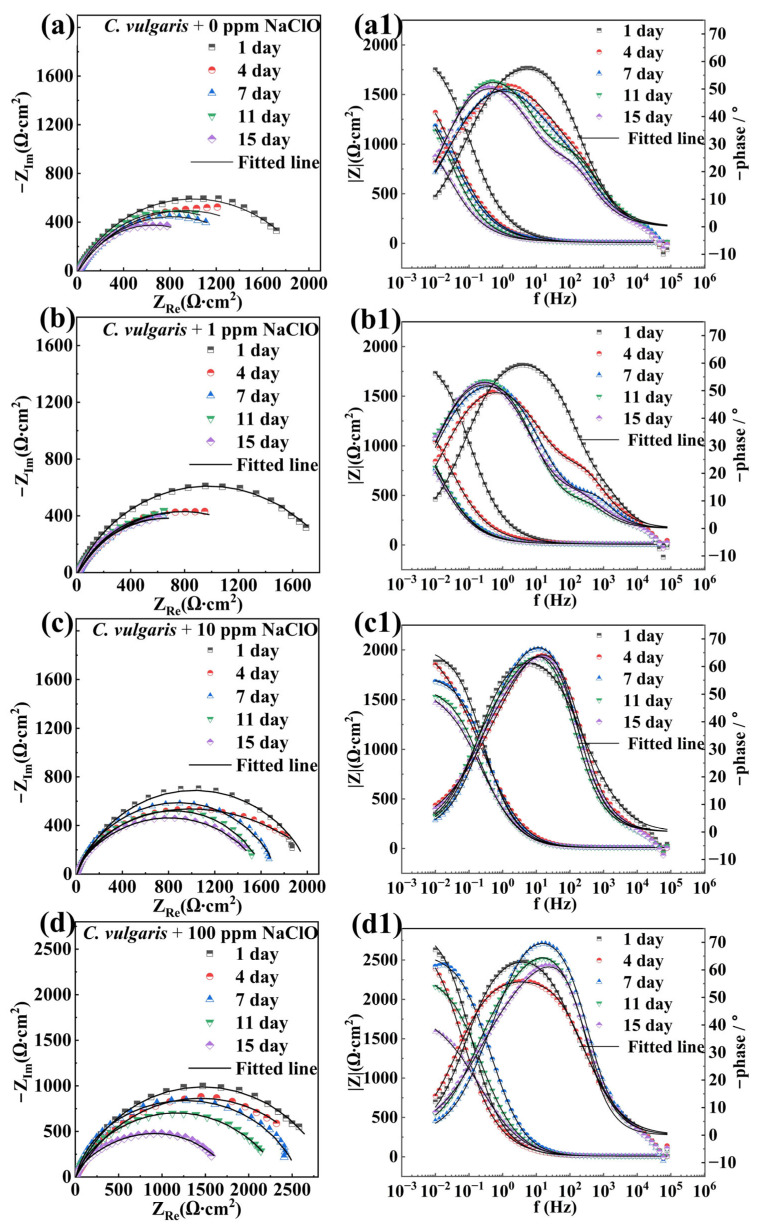
Nyquist and Bode diagrams of the specimens in the biotic solution under the condition of (**a**,**a1**) 0 ppm, (**b**,**b1**) 1 ppm, (**c**,**c1**) 10 ppm, and (**d**,**d1**) 100 ppm NaClO.

Figure 2, Figure 3 and Figure 4 present the continuously monitored OCP values and EIS spectra of 45# steel under different NaClO conditions during the 15-day experimental period. As shown in Figure 2a, the OCP values of the specimens in the abiotic solution exhibited a continuous decreasing trend throughout the experiment at low NaClO concentrations (0, 1, and 10 ppm), likely due to ongoing corrosion. However, at high NaClO concentration (100 ppm), the specimens showed an initial decrease followed by an increase in the OCP, possibly resulting from severe corrosion and substantial accumulation of corrosion products on the surface. The variation trend of OCP values for the 45# steel in the biotic solution was essentially equal to that observed in the abiotic solution (Figure 2b). Comparative analysis of Figure 2a,b reveals that specimens in the biotic solution displayed higher OCP values than those in the abiotic solution, with these differences potentially attributable to the life activity of the algae.

**Figure 5:**
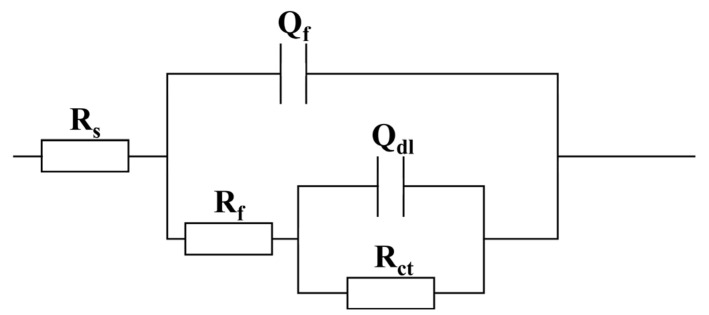
Equivalent circuits used for curve-fitting of EIS spectra.

As shown in Figure 3(a1–d1) and Figure 4(a1–d1), the Bode plots of specimens in both abiotic and biotic solutions exhibited a relatively wide peak or two distinct peaks. As can be seen from the Bode plots, the impedance value of the sample in the abiotic solution decreased with increasing NaClO concentration, indicating that NaClO could reduce the corrosion resistance of carbon steels. In the biotic solution, the impedance value of the sample increased with increasing NaClO concentration, suggesting that NaClO could inhibit corrosion induced by *C. vulgaris*. The equivalent circuit model presented in Figure 5 is employed to fit the EIS spectra of specimens in abiotic and biotic solutions, where *R*_s_ represents the resistance of the test solution, *Q*_f_ and *R*_f_ denote the capacitance and resistance of corrosion product film or biofilm, respectively, and *Q*_dl_ and *R*_ct_ correspond to the capacitance of the electric double layer and charge transfer resistance, respectively. The relevant parameters are listed in Table 1 and Table 2. Replace capacitance (*C*) with the constant phase element (*Q*). The impedance of *Q*_f_ and *Q*_dl_ can be calculated by Equation (1):

(1) $$Z_{\mathrm{CPE}}=Y_{0}^{-1}{\left(j\omega \right)}^{-n}$$

where *Y*_0_ and *n* are *CPE* parameters related to capacitance and surface heterogeneity, and *j* and *ω* are the imaginary root and the angular frequency, respectively.

**Figure 6:**
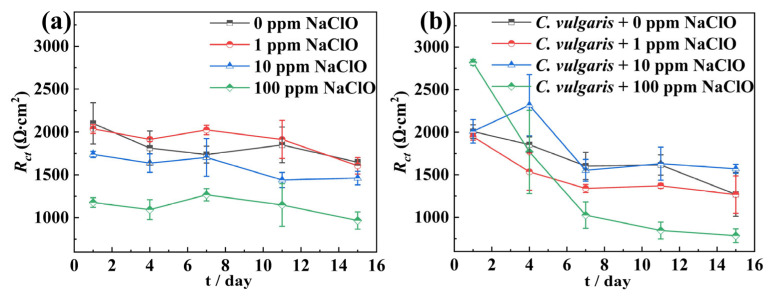
The change in the *R*_ct_ over time in the abiotic solution (**a**) and the biotic F/2 solution (**b**) during the 15-day experimental period.

In the abiotic solution, the *R*_ct_ of specimens exhibited a decreasing trend with increasing NaClO concentration, indicating that NaClO accelerated its corrosion (Figure 6a). In contrast, the biotic solution showed different corrosion behavior (Figure 6b). Overall, compared to the 0 ppm NaClO, the 1 ppm NaClO caused a slight decrease in the *R*_ct_ of the specimen, while 10 ppm NaClO resulted in increased *R*_ct_ values relative to 0 ppm and 1 ppm NaClO, suggesting this concentration effectively inhibited *C. vulgaris*-induced corrosion. At 100 ppm NaClO, the *R*_ct_ of specimens in the biotic solution was higher than that of other NaClO concentrations in the first 4 days, likely due to substantial accumulation of corrosion products on the surface, while after 4 days, the *R*_ct_ value of the specimens decreased significantly, being relatively consistent with that in the abiotic solution. In the biotic solution, the specimens exhibited relatively high *R*_f_ values at 100 ppm NaClO, reaching a particularly significant *R*_f_ value of 1552 Ω on the 7th day, confirming the deposition phenomenon of corrosion products or algal metabolites on the surface as mentioned above. Furthermore, throughout the 15-day experiment, the *R*_ct_ value of 45# steel in both abiotic and biotic solutions displayed a decreasing trend, demonstrating a gradual decline in the corrosion resistance over time. 

**Figure 7:**
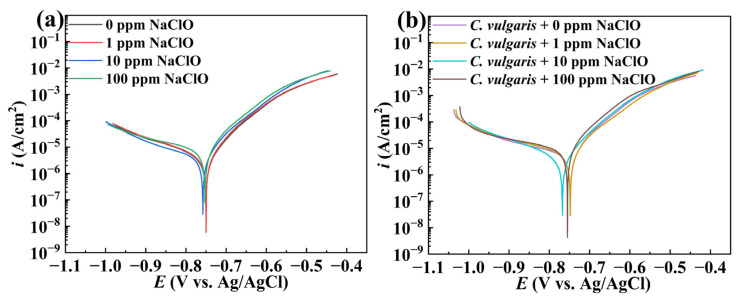
Potentiodynamic polarization curves of 45# steel specimens after 15 days of immersion in the (**a**) abiotic and (**b**) biotic solutions.

After 15-day immersion in both abiotic and biotic solutions, the potentiodynamic polarization curves of 45# steel under different NaClO conditions are measured, as shown in Figure 7. In both abiotic and biotic solutions, the anodic reactions of specimens were accelerated with increasing NaClO concentration. Linear fitting was performed on the anode and cathode regions of the strongly polarized area of polarization curves, respectively. The potential corresponding to the intersection point of the anode and cathode fitting lines was the corrosion potential, and the corresponding current density was the corrosion current density. The relevant parameters are listed in Table 3. As shown in Table 3, the corrosion potentials of specimens showed minimal variation across different NaClO concentrations in the abiotic and biotic solutions. In the abiotic solution, the corrosion current density of the specimen increased with the NaClO concentration, demonstrating the accelerating effect of NaClO on the corrosion of carbon steels. In the biotic solution, the specimens at 0 ppm and 1 ppm NaClO exhibited similar corrosion current densities, while at 10 ppm NaClO, the corrosion current density decreased compared to 0 ppm and 1 ppm NaClO, indicating the inhibitory effect of NaClO on *C. vulgaris*-induced corrosion. However, at 100 ppm NaClO, the corrosion current density increased significantly compared to other concentrations. Notably, the specimens in the biotic solution consistently displayed higher corrosion current density than those in the abiotic solution, suggesting that even after complete algal eradication by 100 ppm NaClO, residual metabolic products continued to accelerate the corrosion of carbon steels.

### 2.3. Weight Loss

**Figure 8:**
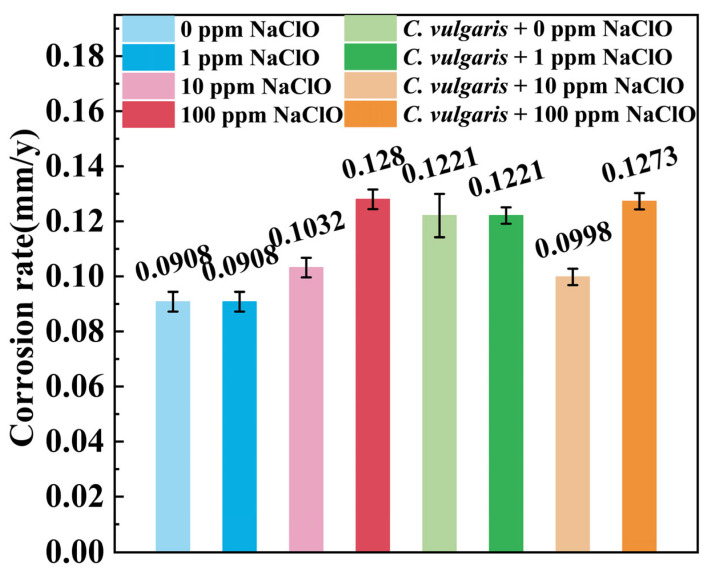
The corrosion rates of specimens calculated from weight-loss tests after 15-day immersion under different NaClO concentrations in the abiotic (0 ppm, 1 ppm, 10 ppm, and 100 ppm NaClO) and biotic solutions containing *C. vulgaris* (0 ppm, 1 ppm, 10 ppm, and 100 ppm NaClO).

To visually assess the coupled effects of NaClO and *C. vulgaris* on the corrosion of carbon steels, the average corrosion rates are calculated through weight-loss measurements. Figure 8 shows the corrosion rates of specimens after 15-day immersion in both abiotic and biotic solutions under different NaClO concentrations. Under the condition of 0 ppm NaClO, the average corrosion rate of the specimen in the abiotic solution was 0.0908 mm/y. The addition of 1 ppm NaClO had a negligible effect on the corrosion rate, while the addition of 10 ppm and 100 ppm NaClO increased the corrosion rate of the specimen to 0.1032 mm/y and 0.128 mm/y, respectively. Compared to the abiotic solution (0.0908 mm/y), the specimen in the biotic solution at 0 ppm NaClO exhibited an elevated corrosion rate of 0.1221 mm/y. Similar to the abiotic solution, 1 ppm NaClO in the biotic solution showed minimal impact on the corrosion rate. However, after the addition of 10 ppm NaClO, the corrosion rate of the specimen in the biotic solution decreased slightly (0.0998 mm/y) compared to that in the abiotic solution. This is due to the interaction between NaClO and *C. vulgaris*. NaClO is consumed to kill *C. vulgaris* cells, resulting in the weakening of both their corrosive effects on the specimens. When NaClO concentration reached 100 ppm, complete algal eradication occurred within 15 min, shifting the corrosion mechanism to NaClO-induced oxidation alone, which explained the nearly identical corrosion rate observed in both abiotic and biotic solutions at 100 ppm NaClO.

### 2.4. Macroscopic and Microscopic Morphologies of Specimen Surfaces

**Figure 9:**
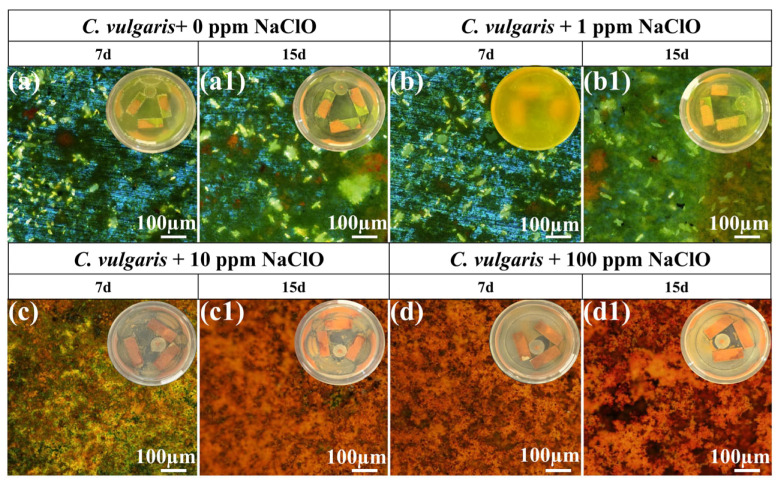
Macroscopic morphologies of surface biofilms and corrosion products after immersion for 7 days under the NaClO concentration of (**a**) 0 ppm, (**b**) 1 ppm, (**c**) 10 ppm, and (**d**) 100 ppm, and for 15 days under the NaClO concentration of (**a1**) 0 ppm, (**b1**) 1 ppm, (**c1**) 10 ppm, and (**d1**) 100 ppm in the biotic solution.

The corrosion of carbon steels by *C. vulgaris* is primarily attributed to algal adhesion on the substrate surface, which significantly influences the corrosion process. Figure 9a–d presents macroscopic images of specimen surfaces after 7 and 15 days of immersion. Under 0 ppm and 1 ppm NaClO conditions, the specimen surfaces were partially covered by *C. vulgaris* biofilms, with remaining areas covered by red iron oxides. The scratches left by polishing could still be observed in the areas covered by the continuous biofilm, while severe corrosion occurred in the areas with discontinuous biofilm. At 10 ppm NaClO, *C. vulgaris* biofilm remained observable after 7 days but was completely replaced by red iron oxides after 15 days. With 100 ppm NaClO treatment, complete inactivation of *C. vulgaris* cells led to uniform surface coverage by red iron oxides.

**Figure 10:**
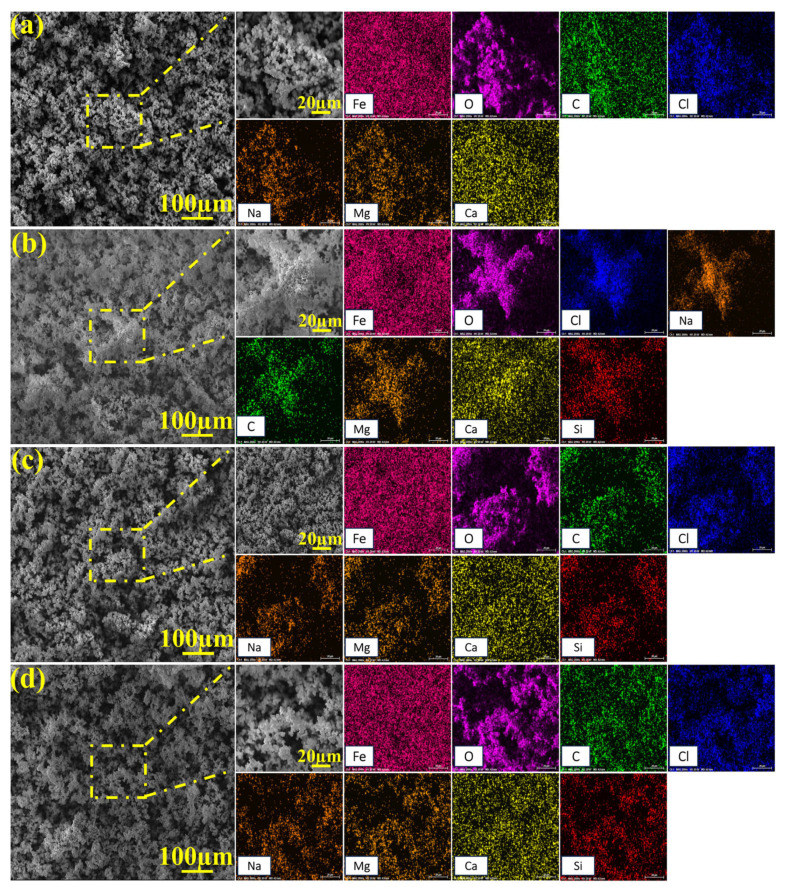
SEM images of corrosion products on specimen surfaces after 15-day immersion under the NaClO concentration of (**a**) 0 ppm, (**b**) 1 ppm, (**c**) 10 ppm, and (**d**) 100 ppm in the abiotic solution.

**Figure 11:**
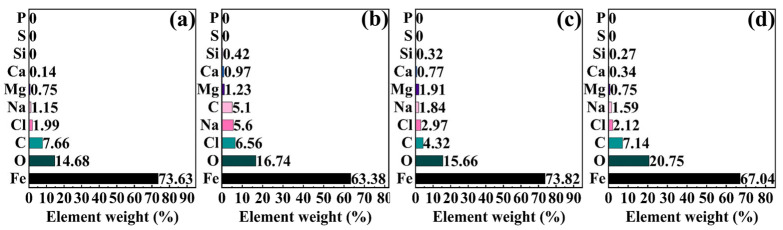
EDS analysis of corrosion products on specimen surfaces after 15-day immersion under the NaClO concentration of (**a**) 0 ppm, (**b**) 1 ppm, (**c**) 10 ppm, and (**d**) 100 ppm in the abiotic solution.

Figure 10 and Figure 11 present the SEM images and EDS mapping results of corrosion product films formed on 45# steel in the abiotic solution, respectively. As shown in Figure 10, the corrosion products exhibited loose and porous morphologies under all NaClO concentrations, demonstrating essentially no protective effect on the substrate. The EDS results in Figure 11 reveal that the corrosion product films primarily consisted of Fe, O, C, Cl, Na, Mg, Ca, and Si elements under different conditions.

**Figure 12:**
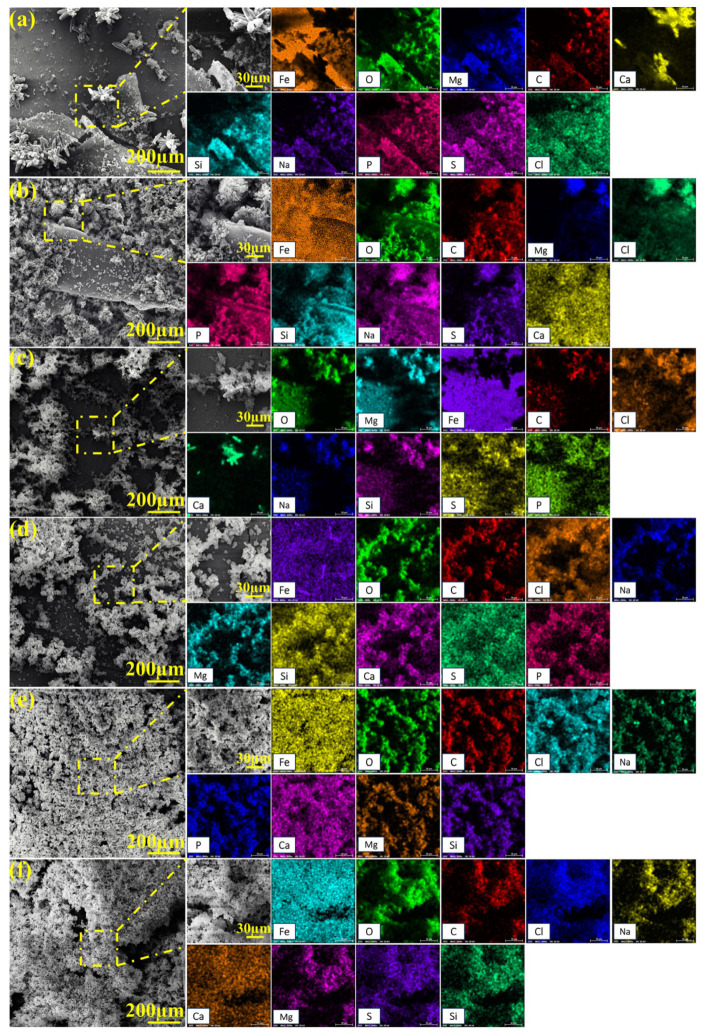
SEM images of biofilms and corrosion products on specimen surfaces after 15-day immersion under the NaClO concentration of 0 ppm for (**a**) biofilm-covered areas and (**b**) biofilm-discontinuous areas, 1 ppm for (**c**) biofilm-covered areas and (**d**) biofilm-discontinuous areas, (**e**) 10 ppm and (**f**) 100 ppm in the biotic solution.

**Figure 13:**
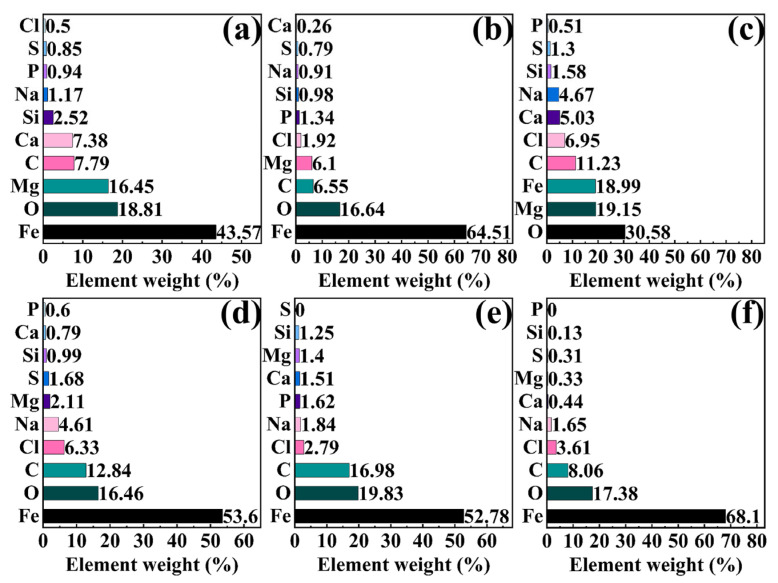
EDS analysis of biofilms and corrosion products on specimen surfaces after 15-day immersion under the NaClO concentration of 0 ppm for (**a**) biofilm-covered areas and (**b**) biofilm-discontinuous areas, 1 ppm for (**c**) biofilm-covered areas and (**d**) biofilm-discontinuous areas, (**e**) 10 ppm and (**f**) 100 ppm in the biotic solution.

After 15-day immersion in the biotic solution, 45# steel surfaces developed distinct corrosion product morphologies and element composition for different NaClO conditions, as shown in Figure 12 and Figure 13. Under 0 ppm and 1 ppm NaClO conditions, the specimen surface in the biotic solution was divided into biofilm-covered areas and biofilm-discontinuous areas, with significant differences in the product morphology between the two areas. In the biofilm-covered areas, the EPS film produced by *C. vulgaris* was relatively dense, mainly composed of elements O, Mg, Fe, C, and Ca, which could effectively prevent the corrosion of the substrate. At 10 ppm and 100 ppm NaClO concentrations, complete biofilm eradication occurred after the 15-day exposure, resulting in uniform surface coverage by porous corrosion products with the composition of O, Fe, and C elements, similar to the biofilm-discontinuous areas observed at lower NaClO concentrations.

### 2.5. XPS Analysis Results

**Figure 14:**
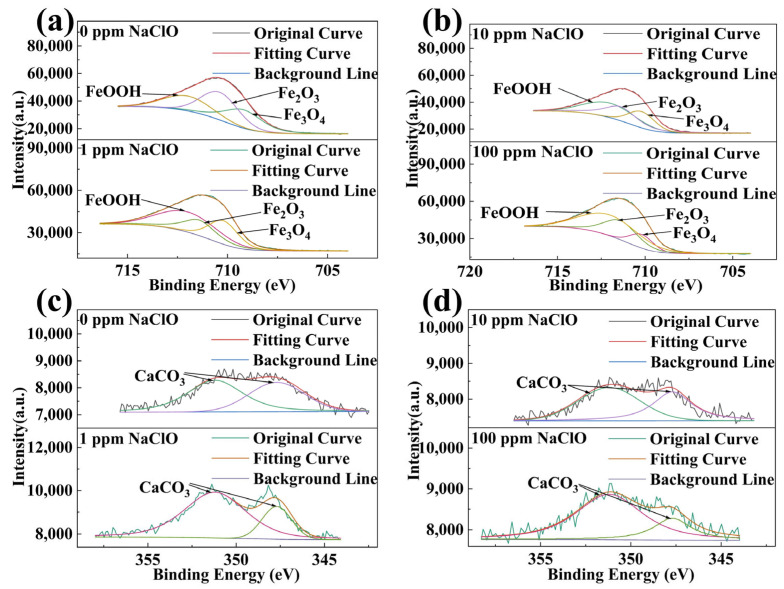
High-resolution XPS spectra of (**a**,**b**) Fe 2p and (**c**,**d**) Ca 2p for 45# steel in the abiotic solution.

**Figure 15:**
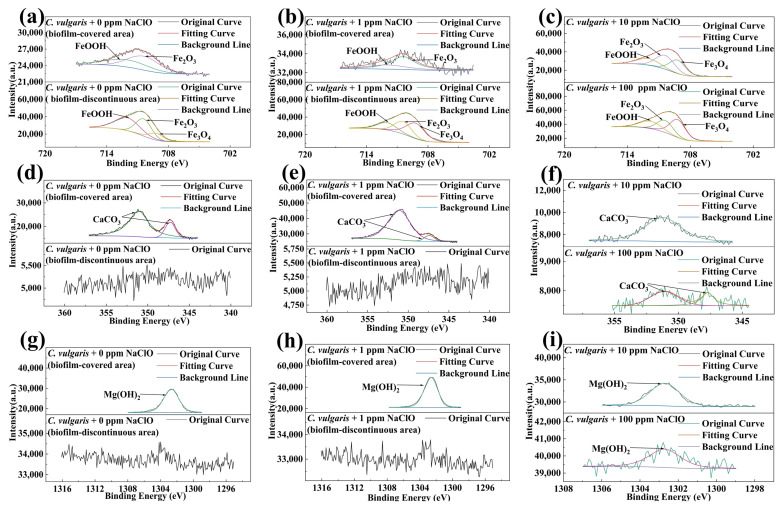
High-resolution XPS spectra of (**a**–**c**) Fe 2p, (**d**–**f**) Ca 2p, and (**g**–**i**) Mg 1s for 45# steel in the biotic solution.

To further determine the composition of corrosion products, XPS tests were conducted to examine the elemental valence states of surface corrosion product on 45# steel specimens after 15-day immersion in both abiotic and biotic solutions, as shown in Figure 14 and Figure 15. The Fe 2p spectra of 45# steel in both abiotic and biotic solutions could be decomposed into FeOOH (≈712.0 eV), Fe_2_O_3_ (≈710.4 eV and 711.4 eV), and Fe_3_O_4_ (≈709.2 eV and 710.2 eV) (Figure 14a,b and Figure 15a–c), indicating that *C. vulgaris* did not change the composition of Fe corrosion products on the specimen surface. The Ca 2p spectra of the specimens in the abiotic solution were mainly decomposed into CaCO_3_ (Figure 11c,d), while in the biotic solution, the Ca 2p spectra in the biofilm-covered areas on the specimen surface showed a CaCO_3_ peak, with no CaCO_3_ detected in the biofilm-discontinuous areas under 0 ppm and 1 ppm NaClO concentrations. For 10 ppm and 100 ppm NaClO conditions, weak CaCO_3_ peaks were detected on the specimen surface, with peak intensities close to CaCO_3_ peaks in the abiotic solution. This demonstrates that *C. vulgaris* biofilm (containing EPS and other substances) could promote CaCO_3_ deposition on specimen surfaces. In the biotic solution, Mg 1s spectra under different NaClO concentrations could be deconvoluted into Mg(OH)_2_ (≈1302.7 eV), with Mg(OH)_2_ exclusively present in biofilm-covered areas at 0 ppm and 1 ppm NaClO, while being undetectable in biofilm-discontinuous areas.

### 2.6. Corrosion Morphology After Removing Corrosion Products

**Figure 16:**
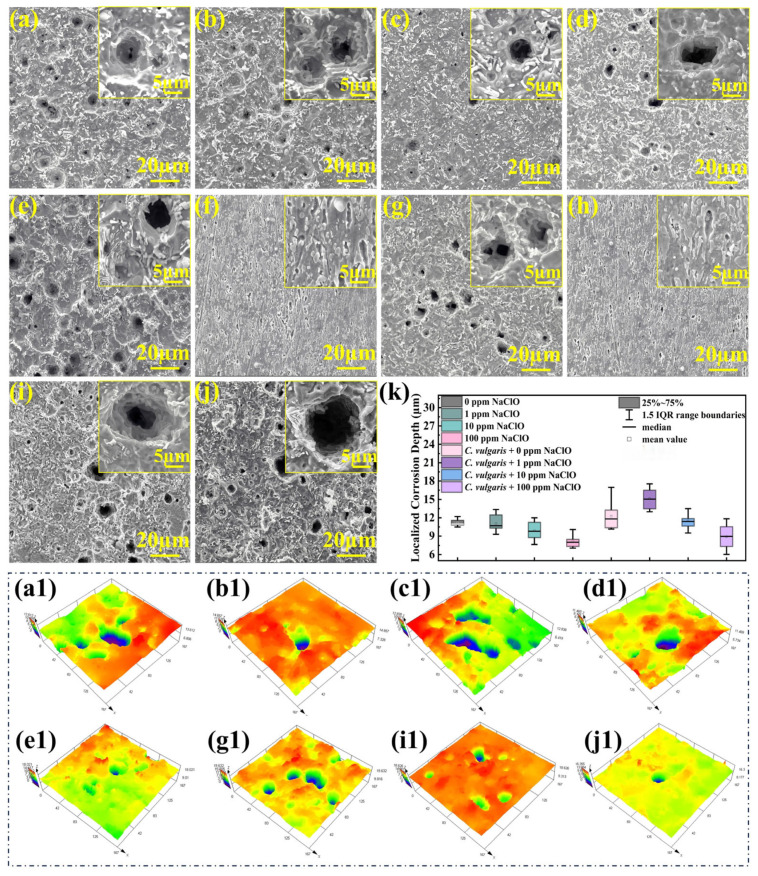
SEM images and 3D color topographic maps of the specimen surfaces after the removal of corrosion products in the abiotic solution containing (**a**,**a1**) 0 ppm NaClO, (**b**,**b1**) 1 ppm NaClO, (**c**,**c1**) 10 ppm NaClO, and (**d**,**d1**) 100 ppm NaClO, and in the biotic solution containing 0 ppm NaClO for (**e**,**e1**) biofilm-discontinuous areas and (**f**) biofilm-covered areas, 1 ppm NaClO for (**g**,**g1**) biofilm-discontinuous areas and (**h**) biofilm-covered areas, (**i**,**i1**) 10 ppm and (**j**,**j1**) 100 ppm, and (**k**) the statistics of localized corrosion depth.

Figure 16 presents the SEM images and 3D color topographic maps of the specimens after 15-day immersion following the removal of corrosion products, with localized corrosion occurring on specimen surfaces in both abiotic and biotic solutions across all NaClO concentrations. In abiotic solutions, the corrosion product film formed on the steel surface was relatively loose and porous. The areas not covered by the product film could act as anodes, while the covered areas could serve as cathodes, leading to the anodic dissolution of the exposed substrate and resulting in localized corrosion. Under the conditions of 0 ppm and 1 ppm NaClO, the corrosion damage in the biofilm-covered areas (Figure 16f,h) on the specimen surface in the biotic solution was much less severe than that in the biofilm-discontinuous areas (Figure 16e,g), with obvious scratches still observable. This indicates that the biofilms (containing EPS and CaCO_3_) have a certain inhibitory effect on the corrosion of carbon steels. To quantify the corrosion severity, the corrosion penetration depths on the specimen surfaces in abiotic and biotic solutions were systematically measured. For each condition, 12 depth data points were collected, with the maximum and minimum outliers excluded, as shown in Figure 16k. At 0 ppm and 1 ppm NaClO, the corrosion penetration depth of the biofilm-discontinuous areas in the biotic solution was deeper than that in the abiotic solution, while there was no obvious localized corrosion in the biofilm-covered areas. At 10 ppm NaClO, the specimen in the biotic solution showed only slightly greater localized corrosion than that in the abiotic solution. This could be attributed to the fact that although *C. vulgaris* existed on the specimen surface after 15 days (Figure 1a), no complete biofilm was formed (Figure 9(c1)), and algal populations were reduced, resulting in mild algae-induced corrosion. At 100 ppm NaClO, the corrosion penetration depth on the specimen surface in the biotic solution was relatively close to that in the abiotic solution, which was consistent with the weight-loss data. In addition, it can be seen from Figure 16k that the increase in NaClO concentration in the abiotic solution reduced the average localized corrosion depth on the specimen surface, indicating that NaClO preferentially promotes uniform corrosion rather than localized corrosion of carbon steels.

### 2.7. The Corrosion Mechanism of Carbon Steels Induced by C. vulgaris and NaClO

**Figure 17:**
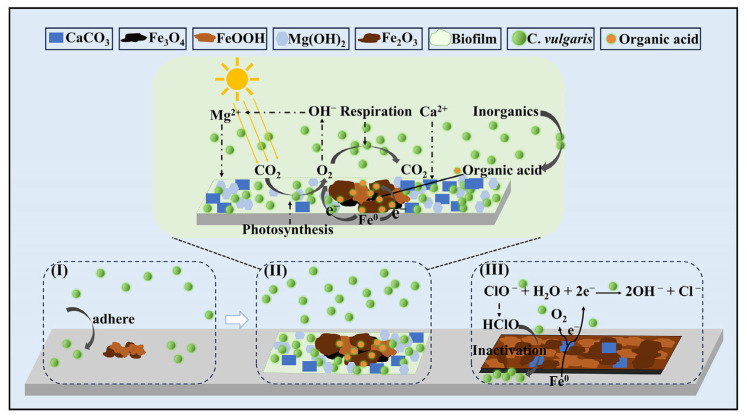
Schematic diagram of corrosion process on 45# steel induced by *C. vulgaris* and influenced by NaClO at this stage of I: initial attachment of algae, II: the formation of algal biofilms and surface corrosion product films and III: the influence of high-concentration NaClO on algae-induced corrosion.

Based on the above experimental results and analyses, a possible corrosion mechanism of 45# carbon steel induced by *C. vulgaris* influenced by NaClO is proposed, as shown in Figure 17. When the steel specimen is immersed in the artificial seawater, the oxidation of Fe^0^ first occurs on the surface, accompanied by the cathodic oxygen reduction. The redox reactions are as follows:

Anodic reaction (oxidation of Fe^0^):

(2) $${\mathrm{Fe}}^{0}\;\to \;{\mathrm{Fe}}^{2+}\;+\;2e^{-}$$

Cathodic reaction (oxygen reduction):

(3) $$O_{2}\;+\;H_{2}O\;+\;4e^{-}\;\to \;4{\mathrm{OH}}^{-}$$

The dissolved Fe^2+^ can combine with OH^−^ to form Fe(OH)_2_, which can be further oxidized by dissolved O_2_ to FeOOH [27]:

(4) $${\mathrm{Fe}}^{2+}\;+\;{\mathrm{OH}}^{-}\;\to \;\mathrm{Fe}{\left(\mathrm{OH}\right)}_{2}$$

(5) $${4\mathrm{Fe(OH)}}_{2}+O_{2}\;\to \;4\mathrm{FeOOH}+2H_{2}O$$

The FeOOH is unstable and will continue to decompose into Fe_2_O_3_ and Fe_3_O_4_ [28,29]:

(6) $$2\mathrm{FeOOH}\;\to \;{\mathrm{Fe}}_{2}O_{3}\;+\;H_{2}O$$

(7) $$8\mathrm{FeOOH}+{\mathrm{Fe}}^{2+}+{2e}^{-}\;\to \;3{\mathrm{Fe}}_{3}O_{4}+4H_{2}O$$

When *C. vulgaris* is introduced into the artificial seawater, algal cells will migrate to the metal surface. Subsequent physical adsorption or chemical bonding facilitates the attachment of the algal cells to the substrate steel (process I). More algal cells progressively adhere to the steel surfaces over time to form biofilms primarily composed of EPS and inorganic precipitates (process II). The respiratory activity of *C. vulgaris* will generate CO_2_, promoting CaCO_3_ deposition within the biofilms. Meanwhile, the *C. vulgaris*-containing solution maintains a pH value around 10, facilitating the formation of Mg(OH)_2_ on the metal surface. The formation processes of CaCO_3_ and Mg(OH)_2_ are as follows:

(8) $${\mathrm{Mg}}^{2+}\;+\;2{\mathrm{OH}}^{-}\;\to \;{\mathrm{Mg}(\mathrm{OH})}_{2}$$

(9) $${\mathrm{Ca}}^{2+}+{\mathrm{OH}}^{-}+{\mathrm{HCO}}_{3}^{-}\;\to \;\mathrm{Ca}{\mathrm{CO}}_{3}+H_{2}O$$

Therefore, the biofilm mainly comprises EPS, CaCO_3,_ and Mg(OH)_2_. It has a certain barrier effect on the corrosion of the substrate carbon steel, which may be due to the fact that this biofilm can prevent the entry of corrosive ions in artificial seawater and the migration of the dissolved cations (Fe^2+^ and Fe^3+^) away. At the same time, the corrosion product films in the biofilm-discontinuous areas are relatively loose, permitting the permeation of aggressive ions (e.g., Cl^−^), and *C. vulgaris* cells can generate a large amount of organic acids and O_2_ through photosynthetic reaction. These species can easily diffuse to the areas not perfectly covered by the biofilms to synergistically exacerbate the localized corrosion at these sites. When a high concentration of NaClO (particularly 100 ppm) is introduced, *C. vulgaris* cells are essentially eradicated, and the corrosion of carbon steels is dominated by NaClO. In this case, in addition to the oxygen reduction reaction, the cathodic reaction also includes the reduction of ClO^−^ (process III):

(10) $${\mathrm{ClO}}^{-}\;+\;H_{2}O\;+\;{2e}^{-}\;\to \;{\mathrm{Cl}}^{-}\;+\;{2\mathrm{OH}}^{-}$$

In summary, a too low concentration of NaClO (0 ppm and 1 ppm) cannot inhibit the growth of *C. vulgaris* and its biocorrosion. Although 100 ppm NaClO can completely kill *C. vulgaris*, it will severely accelerate the corrosion of carbon steels. Therefore, the dosage of NaClO must be carefully controlled in practical applications.

## 3. Materials and Methods

### 3.1. Experimental Materials

This study employed 45# steel coupons (10 mm × 10 mm × 10 mm) with the following chemical composition (wt%): C 0.45, Si 0.22, Mn 0.72, S 0.02, P 0.03, Cr 0.20, Cu 0.18, Ni 0.20, and Fe balance. Only one surface (1 cm^2^) of each specimen was exposed to the test solution, while the remaining areas were encapsulated with epoxy resin. A copper wire was welded to each sample to establish an electrical connection for electrochemical measurements. For weight-loss measurements, the specimens of 40 mm × 15 mm × 10 mm were used, with only one surface (40 mm × 15 mm) exposed to the test solution and the remaining areas sealed with waterproof tape. Prior to each measurement, the specimens were mechanically ground using silicon carbide paper (180, 240, 400, 600, 800, and 1000 grit), followed by ultrasonic cleaning with pure ethanol for 10 min and UV irradiation for 20 min for drying and sterilization before use.

### 3.2. Algal Cultivation and Inoculation

The simulated seawater used in this study, according to the ASTM D1141-98 standard [30], had the following chemical composition: 24.53 g NaCl, 5.20 g MgCl_2_, 4.09 g Na_2_SO_4_, 1.16 g CaCl_2_, 0.695 g KCl, 0.201 g NaHCO_3_, 0.101 g KBr, 0.027 g H_3_BO_3_, 0.025 g SrCl_2_, 0.003 g NaF, and 1000 mL deionized water. The *C. vulgaris* employed in this study was obtained from Shanghai Guangyu Biological Technology Co., Ltd. (Shanghai, China) The F/2 medium, composed of NaNO_3_ (0.075 g), NaH_2_PO_4_·H_2_O (0.005 g), vitamin solution (1 mL), trace metal solution (1 mL), and simulated seawater (1000 mL) [11], was employed for both *C. vulgaris* cultivation and subsequent biocorrosion experiments. For the abiotic and biotic experiment, the test solutions were the artificial seawater and the F/2 medium, respectively. To simulate natural conditions, the *C. vulgaris* inoculum was cultured under room temperature (23 ± 1 °C) with a light/dark cycle (12:12 h) at an illumination intensity of 2400 lux. The dissolved oxygen (DO) concentrations in test solutions under different conditions were measured using a dissolved oxygen meter. During the 15-day experimental period, the optical density (OD) values at 686 nm wavelength were recorded daily using a spectrophotometer to quantitatively monitor the variation in *C. vulgaris* cell concentration.

### 3.3. Electrochemical Tests

Electrochemical tests were performed with a standard three-electrode system with an Ag/AgCl electrode and a platinum sheet (10 mm × 10 mm × 1 mm) as the reference and counter electrode, respectively. The specimens used as the working electrodes (WE) were connected with the copper wire and embedded in epoxy resin, leaving a surface area of 1 cm^2^. The corrosion potential *E*_corr_, electrochemical impedance spectroscopy (EIS) at the *E*_corr_, and potentiodynamic polarization curve measurements were conducted to estimate the corrosion performance of the 45# carbon steel through an electrochemical workstation (CorrTest, CS310X, Wuhan, China) in the 15-day test period. The EIS and polarization curve experiments started after the *E*_corr_ was stabilized, which typically occurred after 5 min of the *E*_corr_ test. In the EIS test, a sinusoidal voltage with an amplitude of 10 mV (vs. *E*_corr_) was applied in the frequency range from 10^5^ to 10^−2^ Hz, acquiring 9 points per frequency decade. The CS analysis software (Version 6.4) was employed to analyze the EIS and polarization curve data. The potentiodynamic polarization curve test was performed in the potential range from −300 to 300 mV (vs. *E*_corr_) at a scanning rate of 0.167 mV·s^−1^. All the electrochemical tests were repeated at least three times.

### 3.4. Weight-Loss Measurements

After 15 days of exposure, the corrosion products and biofilm were removed from the specimen surfaces using a rust removal solution (500 mL HCl, 500 mL deionized water, and 3.5 g hexamethylenetetramine) [31]. Subsequent cleaning involved sequential ultrasonic treatment in distilled water and anhydrous ethanol, followed by drying in cold air. The weight loss of the specimen was determined using an electronic balance (±1 mg). The corrosion rate (CR) of the specimen was calculated according to the following Equation (11) [32]:

(11) $$\mathrm{CR}\;=\;\frac{87,600(W1-W2)}{\mathrm{\rho St}}$$

where *CR* was the corrosion rate (mm/y), t was the immersion time (h), *W*1 and *W*2 were the weight loss of the specimens (g) before and after the experiment, respectively, *ρ* was the density of carbon steel (g/cm^3^), and *S* was the exposed area of the specimen (cm^2^).

### 3.5. Surface Characterization

The morphology characteristics of corrosion products and biofilms formed on specimen surfaces after 15-day immersion in both abiotic and biotic solutions were examined by scanning electron microscopy (SEM, CEM3000A, CHOTEST, Shenzhen, China). Prior to morphology analysis, the specimens were fixed in the phosphate-buffered saline (PBS) solution containing 4% glutaraldehyde at room temperature for 8 h to maintain biofilm integrity [33]. A graded ethanol series (25%, 50%, 75% and 100% aqueous solutions) was used for progressive dehydration. Elemental composition of the surface films (including corrosion products and biofilms) was determined using energy-dispersive spectroscopy (EDS) coupled with SEM. X-ray photoelectron spectroscopy (XPS) was employed to analyze the chemical states of Fe, Ca, and Mg in the corrosion products and biofilms. Representative regions for XPS analysis were selected based on SEM micrographs, focusing on areas displaying characteristic corrosion features.

The corrosion products were chemically removed using the aforementioned rust removal solution. After sequential rinsing with anhydrous ethanol and drying, the corrosion morphology on the specimen surfaces was examined by SEM again. Additionally, surface corrosion damage was characterized in non-contact mode using a digital microscope (Olympus DSX1000, Tokyo, Japan) to quantify corrosion penetration depth.

## 4. Conclusions

In this paper, the biocorrosion of mild steel 45# or C45 (EN) induced by algae *C. vulgaris* in an artificial seawater and the effect of bactericide NaClO on the biocorrosion are systematically revealed. Based on the obtained experimental results, the following conclusions can be drawn:

(1) In abiotic artificial seawater, bactericide NaClO elevates the overall corrosion rate of the steel but reduces the local corrosion penetration depth because it facilitates uniform oxidation across nearly the entire steel surface.
(2) Algae *C. vulgaris* can adhere to the steel surface to form a biofilm in some areas, protecting the substrate steel from corrosion. However, in the area where the biofilm is discontinuous, localized corrosion is significantly aggravated by the organic acids and O_2_ generated from *C. vulgaris*.
(3) When the concentration of NaClO is too low, e.g., 1 ppm, NaClO does not have a significant inhibitive effect on the growth of *C. vulgaris*, but after the concentration is sufficiently high, e.g., at 10 ppm, the *C. vulgaris*-induced corrosion, including the overall damage and local penetration, in the biotic artificial seawater is obviously inhibited.
(4) If the concentration of NaClO is too high, e.g., at 100 ppm, the *C. vulgaris* in the biotic artificial seawater is completely eradicated. In this case, the corrosion of the steel is mainly governed by NaClO. Consequently, the corrosion rate and corrosion penetration depth in the biotic solution are similar to those in the abiotic solution at 100 ppm NaClO. However, in the abiotic artificial seawater, 100 ppm NaClO can significantly accelerate corrosion.

## Figures and Tables

**Table 1:** The electrochemical parameters derived from the EIS spectra for the 45# steel in the abiotic solution.

Condition	t (d)	*R*_s_ (Ω·cm^2^)	*Q*_f_ × 10^−6^ (S·cm^−2^·s^n^)	*n*_f_	*R*_f_ (Ω·cm^2^)	*Q*_dl_ × 10^−6^ (S·cm^−2^·s^n^)	*n_dl_*	*R*_ct_ (Ω·cm^2^)	χ^2^ (×10^−3^)
0 ppm NaClO	1	8.182	301.6	0.806	50.02	496.3	0.733	2100	1.14
	4	8.904	197.7	0.792	672.3	231.8	0.681	1812	7.57
	7	7.241	219.1	0.862	550.3	527.5	0.522	1736	0.933
	11	10.36	226.4	0.871	315.3	367.1	0.591	1849	1.08
	15	8.325	218.7	0.889	270.2	478	0.469	1647	1.04
1 ppm NaClO	1	6.748	641	0.665	0.1308	19.38	1	2036	3.62
	4	7.117	164.2	0.853	846	328.4	0.672	1914	2.84
	7	7.354	158.5	0.866	978.7	282.6	0.583	2024	4.60
	11	7.998	174.4	0.888	402	295.1	0.524	1913	1.62
	15	9.016	214.8	0.886	522.4	428.9	0.558	1606	8.60
10 ppm NaClO	1	5.384	567.9	0.739	67.03	1196	0.695	1738	2.31
	4	8.634	178.4	0.804	373.3	325.3	0.543	1637	1.62
	7	8.539	145.3	0.794	17.37	45.02	0.644	1703	8.73
	11	8.589	277.2	0.887	572	441.6	0.486	1440	1.46
	15	9.859	327.4	0.832	674.3	452.5	0.624	1462	1.28
100 ppm NaClO	1	16.03	108.7	0.755	61.81	691.9	0.621	1177	1.41
	4	8.69	47.85	0.809	608.599	582	0.574	1094	3.35
	7	9.827	261.9	0.790	433.5	890.3	0.604	1267	13.6
	11	13.15	137.2	0.795	762.2	1482	0.524	1148	86.7
	15	9.885	556.5	0.891	799.5	2280	0.629	967	16.8

**Table 2:** The electrochemical parameters derived from the EIS spectra for the 45# steel in the biotic solution.

Condition	t (d)	*R*_s_ (Ω·cm^2^)	*Q*_f_ × 10^−6^ (S·cm^−2^·s^n^)	*n*_f_	*R*_f_ (Ω·cm^2^)	*Q*_dl_ × 10^−6^ (S·cm^−2^·s^n^)	*n_dl_*	*R*_ct_ (Ω·cm^2^)	χ^2^ (×10^−3^)
*C. vulgaris*	1	7.95	49.03	1	2.965	796.2	0.662	2008	2.1
	4	8.518	194.7	0.868	10.85	1659	0.606	1853	1.83
	7	9.411	567.2	0.7511	22.25	1505	0.609	1603	0.39
	11	7.033	654.5	0.7452	19.92	2084	0.675	1615	1.62
	15	8.379	721	0.7628	17.48	2884	0.661	1269	1.52
*C. vulgaris* + 1 ppm NaClO	1	6.085	40.79	1	3.233	839.8	0.701	1946	2.33
	4	8.433	324.5	0.794	13.47	2359	0.635	1535	1.21
	7	7.566	171.7	0.909	4.147	4545	0.658	1339	0.68
	11	9.779	306.3	0.890	39.24	4776	0.678	1370	1.41
	15	8.519	745.9	0.782	26.636	4689	0.671	1267	1.20
*C. vulgaris* + 10 ppm NaClO	1	7.828	275.3	0.777	2.066	169	0.733	2010	1.53
	4	9.295	231.2	0.856	187.3	655.1	0.475	2040	2.29
	7	8.671	277.6	0.868	224	530.6	0.672	1521	0.69
	11	9.034	343.5	0.857	197	651.3	0.671	1431	1.25
	15	9.82	590	0.797	156.2	1531	0.661	1570	1.58
*C. vulgaris* + 100 ppm NaClO	1	7.232	507.2	0.743	44.97	73.9	0.844	2888	1.67
	4	7.876	634.7	0.718	448.4	265.1	0.638	2412	1.46
	7	7.929	176	0.859	1552	717.3	0.676	1026	1.62
	11	7.822	254.2	0.832	1357.2	429.3	0.577	1013	1.38
	15	7.329	268.4	0.829	942.2	763.3	0.576	869	2.07

**Table 3:** The relative electrochemical parameters of 45# steel specimens calculated from potentiodynamic polarization curves after 15 days of immersion.

Condition	*E*_corr_ (V vs. Ag/AgCl)	*i*_corr_ (μA/cm^2^)	*b*_a_	*b*_c_
0 ppm	−0.750	4.54	74.38	209.17
1 ppm	−0.750	4.93	73.67	218.91
10 ppm	−0.758	5.57	79.13	312.86
100 ppm	−0.752	9.34	77.88	375.39
*C. vulgaris* + 0 ppm	−0.748	7.52	75.10	371.2
*C. vulgaris* + 1 ppm	−0.748	8.65	84.97	263.93
*C. vulgaris* + 10 ppm	−0.768	5.26	77.28	306.83
*C. vulgaris* + 100 ppm	−0.755	11.55	77.34	464.4

## Data Availability

Some or all data, models, or code that support the findings of this study are available from the corresponding author upon reasonable request.
